# 

**Published:** 1884-10

**Authors:** 


WHOLE NUMBER, CCLXIX. OCTOBER, 1884. NUMBER 3, VOLUME XXIV.

### THE BUFFALO Medical and Surgical Journal.

LIBRARY INDEXED S.G.O.

EDITORS:

PUBLISHED BY THE MEDICAL JOURNAL ASSOCIATION.
No. 5 WEST CHIPPEWA ST.
Entered at the Post-Office at Buffalo, N. Y., as Second-class Mail Matter.

